# Non-vitamin K antagonist oral anticoagulants vs. vitamin-K antagonists in patients with atrial fibrillation and chronic kidney disease: a nationwide cohort study

**DOI:** 10.1186/s12959-019-0211-y

**Published:** 2019-11-12

**Authors:** Emma Kirstine Laugesen, Laila Staerk, Nicholas Carlson, Anne-Lise Kamper, Jonas Bjerring Olesen, Christian Torp-Pedersen, Gunnar Gislason, Anders Nissen Bonde

**Affiliations:** 1 Department of Cardiology, Copenhagen University Hospital Herlev and Gentofte, Kildegaardsvej 28, 2900 Hellerup, Denmark; 2 Department of Clinical Medicine, Faculty of Health and Medical Sciences, University of Copenhagen, Blegdamsvej 3B, 2200 Copenhagen, Denmark; 3 The Danish Heart Foundation, Vognmagergade 7, 1120 Copenhagen, Denmark; 4 Department of Nephrology, Copenhagen University Hospital Rigshospitalet, Blegdamsvej 9, 2100 Copenhagen, Denmark; 5 Department of Health Science and Technology, Aalborg University, Fredrik Bajers vej 5, 9100 Aalborg, Denmark; 6 The National Institute of Public Health, University of Southern Denmark, Studiestræde 6, 1455 Copenhagen, Denmark

**Keywords:** Atrial fibrillation, Chronic kidney disease, NOAC, VKA, Oral anticoagulation

## Abstract

**Background:**

We aimed to compare effectiveness and safety of non-vitamin K antagonist oral anticoagulants (NOACs) versus vitamin-K antagonists (VKA) in atrial fibrillation (AF) patients with chronic kidney disease (CKD) not receiving dialysis.

**Methods:**

By using personal identification numbers, we cross-linked individual-level data from Danish administrative registries. We identified every citizen with a prior diagnosis of AF and CKD who initiated NOAC or VKA (2011–2017). An external analysis of 727 AF patients with CKD (no dialysis) was performed to demonstrate level of kidney function in a comparable population. Study outcomes included incidents of stroke/thromboembolisms (TEs), major bleedings, myocardial infarctions (MIs), and all-cause mortality. We used Cox proportional hazards models to determine associations between oral anticoagulant treatment and outcomes.

**Results:**

Of 1560 patients included, 1008 (64.6%) initiated VKA and 552 (35.4%) initiated NOAC. In a comparable population we found that 95.3% of the patients had an estimated glomerular filtration rate (eGFR) < 59 mL/min. Patients treated with NOAC had a significantly decreased risk of major bleeding (hazard ratio (HR): 0.47, 95% confidence interval (CI): 0.26–0.84) compared to VKA. There was not found a significant association between type of anticoagulant and risk of stroke/TE (HR: 0.83, 95% CI: 0.39–1.78), MI (HR: 0.45, 95% CI: 0.18–1.11), or all-cause mortality (HR: 0.99, 95% CI: 0.77–1.26).

**Conclusion:**

NOAC was associated with a lower risk of major bleeding in patients with AF and CKD compared to VKA. No difference was found in risk of stroke/TE, MI, and all-cause mortality.

## Background

Atrial fibrillation (AF) and chronic kidney disease (CKD) often coexist [1], and the number of patients suffering from both conditions is rising globally as a result of an aging population.

The risk of stroke, systemic thromboembolism (TE) and myocardial infarction (MI) is higher among such patients than in AF patients with no renal disease [2, 3]. A Danish study found that AF patients with non-end-stage CKD or end-stage CKD had an increased risk of stroke/TE of 50 and 83%, respectively, compared to patients without renal disease [2]. Furthermore, the presence of both disorders increases the risk of bleeding [2], causing treatment for such patients to be complicated. Oral anticoagulation (OAC) reduces risk of stroke/TE and all-cause mortality in the general AF population [4], and AF patients at high stroke risk are recommended lifelong therapy with a vitamin-K antagonist (VKA) or non-VKA OAC (NOAC). NOACs have proven superior or noninferior to VKA in AF patients in regards of stroke and TE prevention, they are prescribed in fixed dosages, and do not require international normalized ratio (INR) monitoring [5]. Both have also proven effective in reducing the risk of stroke/TE in AF patients with mild to moderate CKD [3, 6], but patients with severe CKD have been excluded from randomized clinical trials of NOAC vs. VKA [5, 7]. All NOACs are dependent on renal clearance [6], which is why there have been concerns regarding efficacy and safety in patients with more advanced CKD.

The aim of this study was to determine whether patients diagnosed with both AF and CKD can benefit from NOAC treatment the same way as patients without CKD can.

## Methods

### Data sources

Every Danish citizen has a unique civil registration number, which allows cross-linkage of several nationwide registries on an individual basis. All discharge diagnoses from Danish hospitals are registered in the nationwide Danish National Patient Registry [8]. Every drug dispensed from a Danish pharmacy is registered in the Danish Prescription Registry [9]. Civil status, yearly income, and cause of death is registered in the Danish Civil Registration System [10].

### Study population

We identified all Danish citizens with a prior diagnosis of AF and CKD who initiated OAC between 22nd August 2011 (the day that the first NOAC, dabigatran, was approved in Denmark) and 30th June 2017 (data in our registries were available until this date). We excluded patients with valvular AF, defined as mechanical heart valve or rheumatic heart disease [11]. Additionally, we excluded patients on dialysis where use of all NOACs are off-label, and patients initiated on edoxaban because of the low number of patients, and due to its short period of time available on the market.

### External analysis of CKD diagnosis

Since plasma creatinine measurements were not available after 2011, we had no data on kidney function in the study population. We estimated kidney function level by studying a comparable CKD population. Thus, we created a dataset of 727 AF patients with CKD (no dialysis) diagnosed between 1997 and 2011who were first-time initiators of OAC. All patients in the dataset had one plasma creatinine measurement within 90 days before initiating OAC. Plasma creatinine values were registered in databases from either a general practitioner or from a hospital clinic. eGFR was calculated using the CKD-EPI equation [12].

### Baseline characteristics

Co-medication and co-morbidity were defined as in earlier studies [13, 14]. In brief, co-medication was identified as prescriptions claimed during the last 180 days before inclusion in the study, and co-morbidity was identified from hospital diagnoses during the last 5 years before inclusion. All ICD and ATC codes used to define our dataset are listed in Additional file 1.

### Outcomes and follow-up

Patients were followed after initiation of OAC until whichever of the following occurred first: emigration, death from any cause, discontinuation/switch of OAC, 30th June 2017, or 1 year after initiation of OAC. We investigated the following events: i) major bleeding, defined as gastrointestinal, urogenital, airway, intraocular or intracranial bleeding causing hospitalization [15], ii) stroke/thromboembolism defined as ischemic or unclassified stroke or systemic arterial thromboembolism [16], iii) myocardial infarction, and iv) all-cause mortality.

### Statistics

Continuous covariates are presented as medians with interquartile range (IQR), and categorical covariates are presented as frequencies with percentages. *P*-value for differences in baseline characteristics were calculated using Wilcoxon-Mann Whitney or Chi square test for continuous or categorical variables, respectively. *P*-value for trend in initiation patterns over time was calculated using the Cochran-Armitage test. Cumulative incidence of event according to OAC was calculated using the Aalen-Johansen estimator, with death considered as competing risk. P-value for difference in cumulative incidence was calculated using Grey’s test. Cox proportional hazards models were used to examine the association between use of OAC and outcome. Models with bleeding as outcome were adjusted for age, sex, prior bleeding, prior stroke, liver disease, hypertension, and use of non-steroid anti-inflammatory drugs, acetylsalicylic acid, adenosine phosphate receptor inhibitors (ADPi), and income during the previous year. Models with stroke/thromboembolism or myocardial infarction as outcome were adjusted for age, sex, prior stroke, heart failure, hypertension, coronary heart disease, diabetes, and use of acetylsalicylic acid, ADPi and income during the previous year. Models with all-cause mortality as outcome were adjusted for age, sex, prior stroke, prior bleeding, coronary heart disease, heart failure, hypertension, diabetes, liver disease, use of non-steroid anti-inflammatory drugs, acetylsalicylic acid, ADPi, and income during the previous year. A Kolmogorov type test was used to examine the proportional hazards assumption, and we tested for interaction between OAC and relevant variables, including age, sex, income, and treatment with ADPi or acetylsalicylic acid in all models. G-formula based on the Cox models were used to calculate standardized absolute risk according to VKA or NOAC treatment each month during follow-up, and 1000 bootstraps calculated confidence intervals. As sensitivity, we calculated hazard ratios (HRs) according to time-varying OAC instead of OAC at baseline, e.g., patients could switch exposure status (switch between VKA and NOAC treatment or vice versa) during follow up. HRs were also calculated according to OAC among AF patients with CKD when not censoring at shift or discontinuation. All analyses were performed with SAS version 9.4, and R version 3.4.1 [17].

## Results

### Study population

**Fig. 1:**
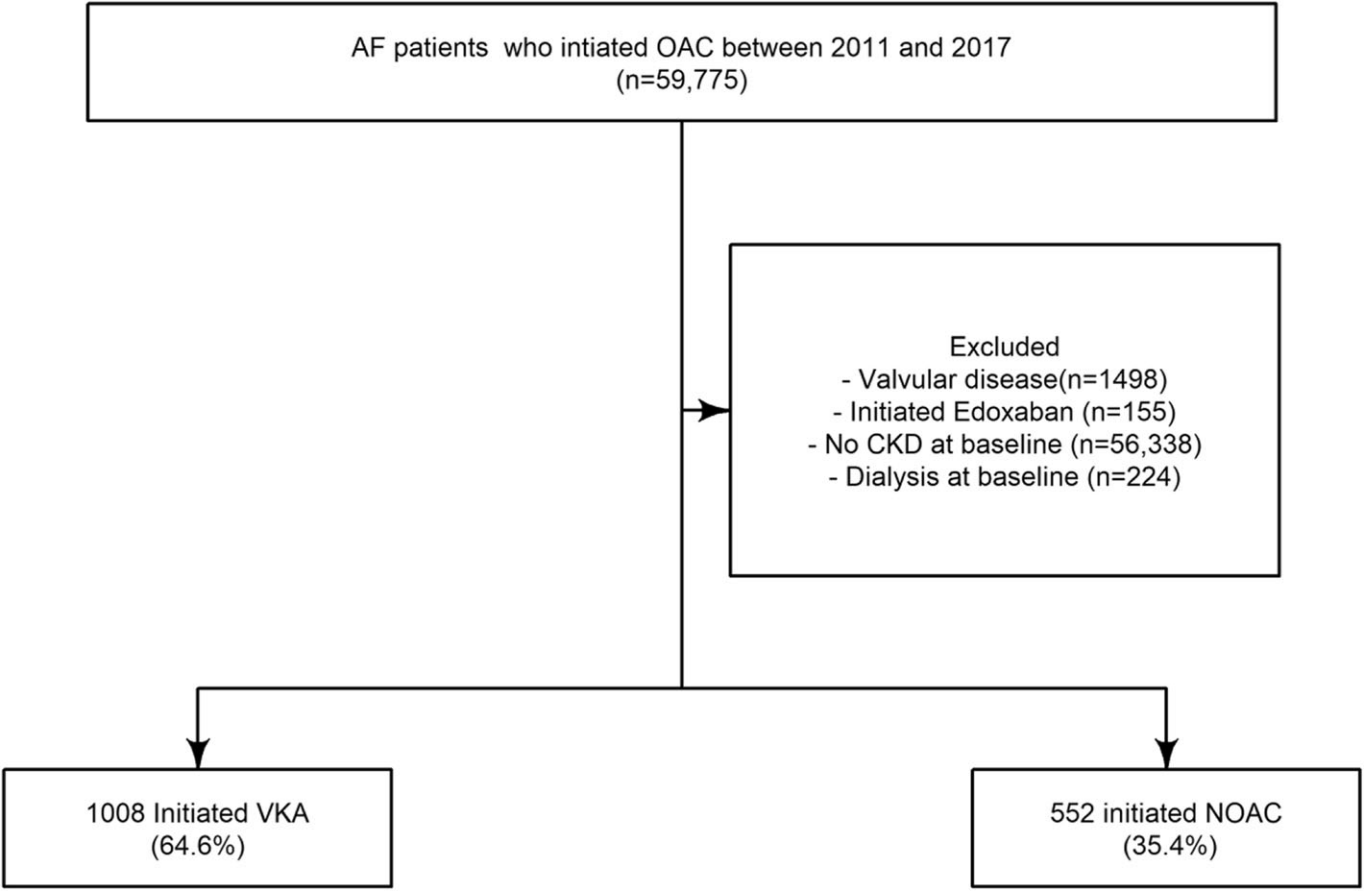
Flowchart of the selection of the study population

A study population of 1560 patients with non-valvular AF and CKD was identified during our study period from 2011 to 2017 **(**Fig. 1**)**. From this population, 1008 (64.6%) patients initiated VKA treatment while 552 (35.4%) initiated NOAC treatment. Of these 552 patients, 302 (54.7%) received apixaban, 170 (30.8%) received rivaroxaban, and 80 (14.5%) received dabigatran. In the NOAC population, 428 patients (77.5%) received reduced dose. The patients who initiated a NOAC were more likely to be older and more often women compared to the VKA patients **(**Table 1**)**. No significant differences were found between the two groups of patients in terms of other co-morbidities or co-medications. Other baseline characteristics are listed in Table 1.

**Table 1:** Baseline characteristics of the study population

	Patients receiving VKA (*n* = 1008)	Patients receiving NOAC (*n* = 552)	*P* value
Median age (IQR)	78.00 (71.00–84.00)	80.00 (72.00–86.00)	< 0.001
Male (%)	645 (64.0)	314 (56.9)	0.007
Oral anticoagulation			< 0.001
Apixaban (%)		302 (54.7)	
Dabigatran (%)		80 (14.5)	
Rivaroxaban (%)		170 (30.8)	
VKA (%)	1008 (100.0)		
Income group (quartiles)			< 0.001
1st (lowest)	382 (37.9)	143 (25.9)	
2nd	297 (29.5)	165 (29.9)	
3rd	208 (20.6)	159 (28.8)	
4th (highest)	121 (12.0)	85 (15.4)	
Comorbidity			
Hypertension	914 (90.7)	494 (89.5)	0.454
Previous stroke (%)	175 (17.4)	102 (18.5)	0.629
Previous bleeding (%)	256 (25.4)	122 (22.1)	0.164
Heart failure (%)	395 (39.2)	195 (35.3)	0.147
Ischemic heart disease (%)	440 (43.7)	231 (42.9)	0.492
Peripheral artery disease (%)	101 (10.0)	63 (11.4)	0.391
Diabetes (%)	260 (25.8)	141 (25.5)	0.962
Liver disease (%)	28 (2.8)	19 (3.4)	0.563
Alcohol abuse (%)	38 (3.8)	31 (5.6)	0.117
Comedication			
ADPi (%)	164 (16.3)	107 (19.4)	0.138
Aspirin (%)	537 (53.3)	277 (50.2)	0.264
Statin (%)	503 (49.9)	275 (49.8)	1.000
Beta-blocker (%)	582 (57.7)	303 (54.9)	0.302
RASi (%)	524 (52.0)	294 (53.3)	0.667
NSAID (%)	116 (11.5)	69 (12.5)	0.619

Abbreviations: *IQR* Interquartile Range, *ADPi* Adenosine diphosphate inhibitor, *VKA* Vitamin K antagonist, *RASi* Renin angiotensin system inhibitor, *NOAC* Nonvitamin K oral anticoagulants, *NSAID* Non-steroid anti-inflammatory drugs

### External analysis of kidney function in a comparable population

In a comparable population of 727 patients with a diagnosis code of AF and CKD, 3 (0.4%) of included patients had an eGFR > 90 mL/min/1.73m^2^, 31 (4.3%) an eGFR 60–90 mL/min/1.73m^2^, 312 (42.9%) an eGFR 30–59 mL/min/1.73m^2^, 319 (43.9%) an eGFR 15-29 mL/min/1.73m^2^, and 62 (8.5%) an eGFR < 15 mL/min/1.73m^2^ (Additional file 2).

In aggregation, 95.3% had an eGFR < 59 mL/min/1.73m^2^ while 52.4% had an eGFR < 29 mL/min/1.73m^2^.

### Risk of stroke/TE, major bleeding, myocardial infarction, and all-cause mortality

**Fig. 2:**
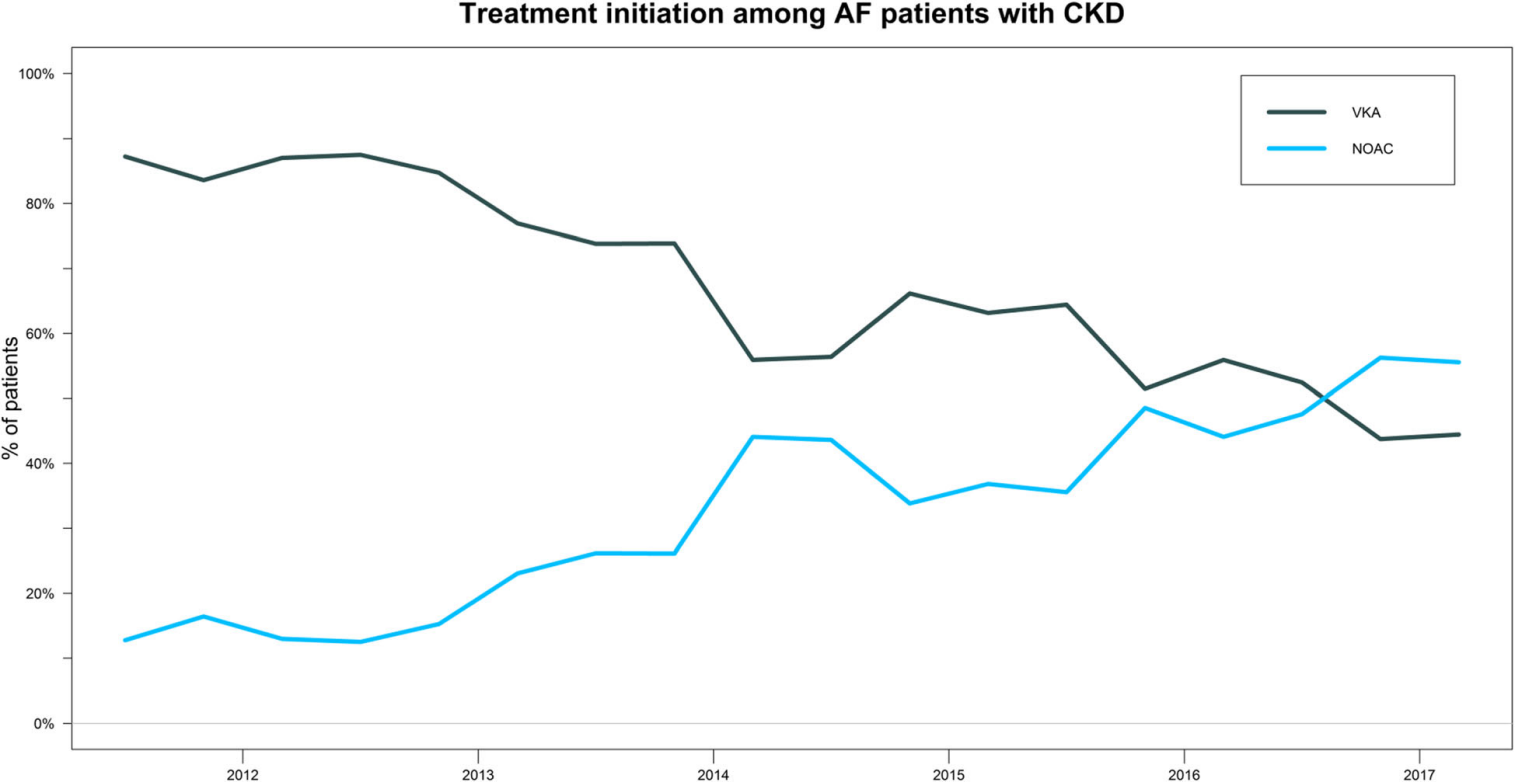
Treatment initiation patterns of OAC among AF patients with CKD between 2011 and 2017

Between 2011 and 2017, a significant increase in initiation of NOAC among AF patients with CKD was observed (*p*-value for trend < 0.001) (Fig. 2). In 2011, 15% initiated a NOAC compared to 60% in late 2017.

Table 2 shows adjusted HRs and 1-year standardized absolute risks of stroke/TE, major bleeding, MI, and all-cause mortality according to type of OAC.

**Table 2:** Risk of events according to OAC in AF and CKD patients

	Number of events	Hazard ratio (95%CI)	Standardized absolute risk (95%CI)
Stroke/thromboembolism			
VKA	21	1.00 (reference)	2.4% (1.4–3.5%)
NOAC	11	0.83 (0.39–1.78)	2.0% (0.8–3.3%)
Major bleeding			
VKA	55	1.00 (reference)	5.9% (4.4–7.5%)
NOAC	15	0.47 (0.26–0.84)	2.8% (1.5–4.3%)
Myocardial infarction			
VKA	22	1.00 (reference)	2.5% (1.5–3.7%)
NOAC	7	0.45 (0.18–1.11)	1.1% (0.4–2.2%)
All-cause mortality			
VKA	183	1.00 (reference)	23.3% (20.2–26.3%)
NOAC	106	0.99 (0.77–1.26)	23.2% (19.4–27.0%)

Abbreviations: *CI* Confidence interval, *OAC* Oral anticoagulation, *VKA* Vitamin-K antagonist, *NOAC* Nonvitamin K oral anticoagulant

The 1-year standardized absolute risk of stroke/TE in AF patients with CKD treated with NOAC was 2.0% (95% confidence interval (CI): 0.8–3.3%) and for the group treated with VKA 2.4% (95% CI: 1.4–3.5%). There was no significant difference between the risk of stroke/TE among NOAC patients compared to VKA patients (HR: 0.83, 95% CI: 0.39–1.78).

**Fig. 3:**
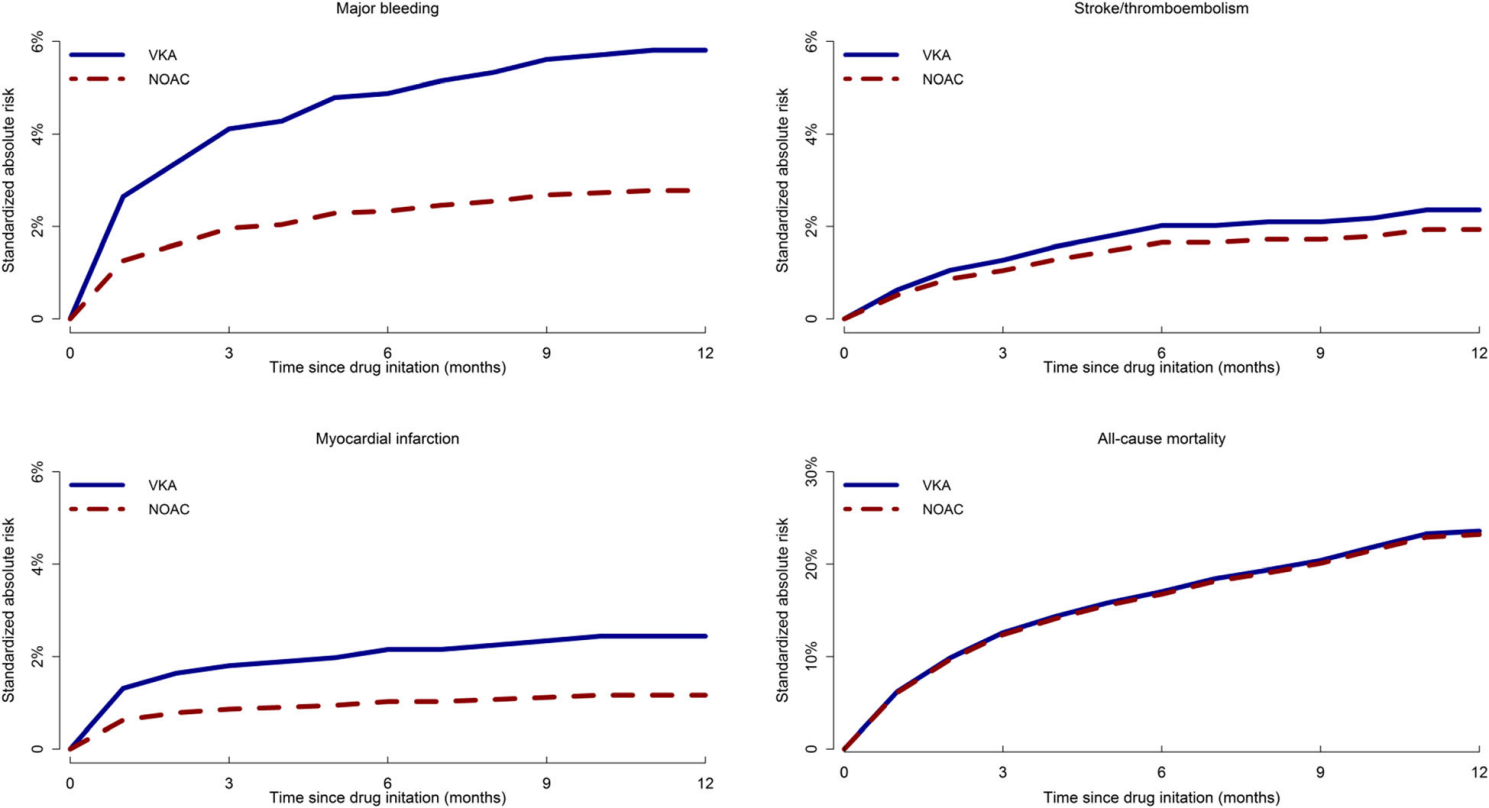
Standardized absolute risk of event according to type of OAC among AF patients with CKD

The 1-year standardized absolute risk of major bleeding in the study population was 2.8% (95% CI: 1.5–4.3%) among NOAC patients and 5.9% (95% CI: 4.4–7.5%) among VKA patients. There was a significant association between major bleeding and type of OAC. Patients receiving a NOAC had a significant lower risk of major bleeding compared to patients on VKAs (HR: 0.47, 95% CI: 0.26–0.84). We found no significant association between use of NOAC and risk of MI (HR: 0.50, 95% CI: 0.21–1.19). The 1-year standardized absolute risk of MI was 1.1% (95% CI: 0.4–2.2%) in the NOAC treated population and 2.5% (95% CI: 1.5–3.7%) in the VKA treated population. The 1-year standardized absolute risk of all-cause mortality was 23.2% (95% CI: 19.4–27.0%) in AF patients with CKD on NOAC and 23.3% (95% CI, 20.2–26.3%) for the ones on VKA. No differences were found in all-cause mortality between the two groups of patients (HR: 0.99, 95% CI: 0.77–1.26). Figure 3 illustrates the standardized absolute risks of stroke/TE, major bleeding, MI, and all-cause mortality, described above, in the first year following drug initiation among patients on NOAC or VKA, respectively. Cumulative incidences of the study outcomes yielded similar results (Additional file 3).

### Sensitivity analyses

Analyses of the study outcomes were repeated with time-varying OAC, allowing patients to change treatment group after inclusion. These results were similar to main analyses (Additional file 4). When not censoring at shift or discontinuation of OAC, outcomes were similar to main results as well **(**Additional file 5**)**.

## Discussion

This nationwide study examined the risk of stroke/TE, major bleeding, MI, and all-cause mortality in AF patients with CKD, comparing patients treated with NOAC to VKA. We had the following important findings: 1) there was a significant gradual increase in the use of NOACs during our study period, 2) NOACs were associated with a significantly lower risk of major bleeding compared to VKA, 3) there were no significant differences in occurrence of MI, stroke/TE or all cause-mortality with NOAC or VKA, and 4) the risk of death of any cause was almost one in four following the first year after treatment initiation among patients with AF and CKD.

### Efficacy and safety of NOAC vs. VKA

VKAs such as warfarin are cleared mainly by the liver [6], but there are no randomized studies comparing warfarin with placebo among patients with moderate or severe CKD. Warfarin-related hemorrhages increase as creatinine clearance decreases [6], and time in therapeutic range with warfarin is often low among patients with CKD, which indicates a need for careful monitoring of these patients or a different treatment strategy. Our study investigated three types of alternatives to warfarin for stroke prevention among AF patients with CKD, specifically apixaban, rivaroxaban, and dabigatran. The renal clearance of these NOACs ranges between 27 and 80% [6], and there has been concern that reduced drug-elimination due to impaired GFR may lead to an increased risk of severe bleedings. Our findings do not support this concern, as NOAC treatment was associated with lower risk of major bleeding compared to VKA in our real-world population.

When looking at NOACs as a group for patients with non-end-stage CKD, our results seem to be in accordance with the existing literature. Effect of renal function on efficacy and safety of NOACs vs. warfarin among AF patients has been studied in the pivotal phase 3 NOAC trials, both among patients with reduced renal function at baseline and among patients with deteriorating renal function after randomization. A review collected data from randomized trials to compare safety and efficacy of NOACs vs. warfarin. They included patients with AF and an eGFR between 15 and 60 mL/min (CKD stages 3 and 4) and concluded that NOACs were just as effective in stroke prevention without causing an increased risk of bleeding, primarily reflecting AF patients with CKD stage 3 [18]. Apixaban reduced risk of bleeding compared to warfarin to a higher degree among patients with renal impairment than among patients without renal impairment [7], whereas dabigatran showed a significant trend towards higher risk of major bleeding compared to warfarin among patients with low renal function compared to patients with normal renal function [19]. The majority of the NOAC-treated study population (54.7%) received apixaban and a minority (14.5%) received dabigatran. Our results could reflect the beneficial safety of apixaban among patients with non-end-stage CKD found in trials, and extent the findings from randomized trials to a real-world population.

Combining results from clinical trials and real-world studies, NOACs have been shown to be as effective and safe as VKA in AF patients with non-end-stage CKD, but all pivotal phase 3 randomized trials of warfarin vs. NOACs excluded patients with creatinine clearance (CrCl) < 25 mL/min [6], and it is still not known whether NOACs are safe and effective among these patients. Renal function data was not available in our study cohort, but by looking at the kidney function distribution in the comparable population, it seems reasonable to hypothesize that a significant number of patients in our study also had an eGFR< 30 mL/min/1.73m^2^.

Our findings are also consistent with a recent retrospective cohort study that investigated the effects of apixaban vs. warfarin in AF patients on dialysis [20]. They found comparable efficacy with apixaban compared to warfarin, but a significant 28% lower HR of major bleeding. The results regarding use of NOACs in end-stage CKD are still hypothesis-generating and ongoing randomized controlled trials will most likely settle these questions.

### Mortality risk among patients with AF and CKD

In our study the risk of dying of any cause was almost one in four following the first year of treatment with NOAC or VKA treatment (23.2% vs. 23.3%). Albeit we found a significant lower risk of major bleeding and a non-significant lower risk of stroke/TE and MI associated with NOAC compared with VKA treatment, we did not find any signals of a difference in the mortality risk between NOAC vs. VKA treatment. Regarding the outcomes major bleeding, stroke/TE, and MI, we adjusted the statistical analyses for competing risk of death, but it was evident that our study population comprising of vulnerable and frail individuals with AF and CKD had a high risk of death, independent of NOAC or VKA treatment. In routine clinical practice, the high risk of death must be taken into account when managing patients with AF and CKD. Also, even though there was no difference in mortality risk between NOAC and VKA treatment, a major bleeding event can be serious or fatal, and the different bleeding risk with NOAC and VKA treatment (2.8% vs. 5.9%) may be considered when initiating OAC treatment among these frail patients.

### Strengths and limitations

In this nationwide study, we included all Danish citizens on OAC with a diagnosis of both AF and CKD. We had practically no loss to follow-up and, the majority of diagnoses used, such as CKD, AF, bleedings, and strokes have been validated [8]. Our study also had limitations. Since creatinine values were not available after 2011, we could not determine exact renal function for each patient in our study, which is a major limitation. For the same reason, we were not able to determine if there was a difference in renal function between the NOAC and VKA population. This limitation makes it difficult to draw definite conclusions from our study and the limitation should be kept in mind when interpreting the results. Further research should be done to establish the effects of NOAC in patients with AF and CKD. The number of study outcomes for stroke/TE and MI are limited, making it uncertain to draw conclusions for those outcomes. The limited number of study outcomes also prevented us from analyzing differences among the individual types of NOAC. Some comorbidities such as hypertension and diabetes were identified using data on prescribed medicine, meaning that patients treated solely with lifestyle changes could not be identified. Also, it is possible that some patients were prescribed this medication for other unknown purposes. The hypertension definition, however, has previously been validated with a positive predictive value of 80% [14]. Finally, as in most observational studies, unmeasured confounding or residual confounding affecting our results cannot be ruled out.

## Conclusion

In patients with AF and CKD, treatment with NOAC was associated with a lower risk of major bleeding, but was not found to either decrease or increase the risk of stroke/TE, MI or all-cause mortality compared to VKA.

## Supplementary information

**Additional file 1.** ICD-8/10 codes and ATC-codes.

**Additional file 2.** External analysis of kidney function in a comparable population.

**Additional file 3.** Cumulative incidence of events according to OAC among AF patients with CKD.

**Additional file 4.** Risk of events according to time-varying OAC.

**Additional file 5.** Hazard ratio according to type of oral anticoagulation among atrial fibrillation patients with CKD when not censoring at shift or discontinuation of oral anticoagulation.

## Data Availability

No additional data are available. Details of statistical analysis are available from the corresponding author on request (emma.laugesen@hotmail.com).

## Abbreviations

ADPi: Adenosine phosphate receptor inhibitors
AF: Atrial fibrillation
ATC: Anatomical therapeutic chemical classification
CI: Confidence interval
CKD: Chronic kidney disease
CrCl: Creatinine clearance
eGFR: Estimated glomerular filtration rate
GFR: Glomerular filtration rate
HR: Hazard ratio
ICD: International classification of disease
INR: International normalized ratio
IQR: Interquartile range
MI: Myocardial infarction
NOAC: Non-vitamin K antagonist oral anticoagulant
NSAID: Non-steroid anti-inflammatory drugs
OAC: Oral anticoagulation
RASi: Renin angiotensin system inhibitor
TE: Thromboembolism
VKA: Vitamin-K antagonists
