# Supplementary material for: Non-vitamin K antagonist oral anticoagulants vs. vitamin-K antagonists in patients with atrial fibrillation and chronic kidney disease: a nationwide cohort study
Source: Thromb J. 2019 Nov 12;17:21. doi: 10.1186/s12959-019-0211-y (PMC6849210; doi:10.1186/s12959-019-0211-y)
Supplement: Supplementary file 1 — Additional file 1. ICD-8/10 codes and ATC-codes. [file 12959_2019_211_MOESM1_ESM.docx]

## Additional file 1: ICD-8/10 codes and ATC-codes

| **Population** |  |  |
| --- | --- | --- |
| Non-valvular atrial fibrillation | *Defined from diagnosis of atrial fibrillation and absence of diagnosis codes of rheumatic valvular disease and mitral- or aortic valve surgery.* | Presence of:  ICD8: 42793, 42794  ICD10: I48  Absence of:  ICD8: 4240, 4241, 39500- 39502, 39508, 39509, 39600-39604, 39608, 39609  ICD10: Z952, Z954, I05, I06, I080A, I081A, I082A, I083A  NCSP: KFKD, KFKH, KFMD, KFMH, KFGE, KFJF |
| Chronic kidney disease | *Defined from diagnosis of chronic kidney disease from hospital* | Presence of:  ICD10: N18  Absence of:  ICD10: N181, N182, N183 |
| Vitamin K antagonist | *Defined from ATC-code* | ATC: B01AA03, B01AA04 |
| Dabigatran | *Defined from ATC-code* | ATC: B01AE07 |
| Rivaroxaban | *Defined from ATC-code* | ATC: B01AF01 |
| Apixaban | *Defined from ATC-code* | ATC: B01AF02 |
|  |  |  |
| **Outcomes** |  |  |
| *Major Bleeding* | *Defined from diagnosis a primary or secondary in-patients diagnosis* | ICD-10: I60, I61, I62, I690, I691,I692, S064,S065 S066,  J942, R04, K228F, K250, K252, K254, K256, K260, K262, K264,K266, K625, K270, K272, K274, K280, K282, K284, K286, K290, K920, *K921,* K922, K228F, K298A, K638B, K638C, K661, K838F, K868G, I850, I864A,N02, R31,  D62, D500, D629,  H113, H356, DH431, H450, H052, S368D, G951A, I312 |
| Stroke/thromboembolism | *Defined from diagnosis of ischemic stroke, transient ischemic attack, or peripheral artery embolism* | ICD10: I63, I64, I74, G458, G459 |
| Myocardial infarction | *Defined from diagnosis a primary in-patient diagnosis* | ICD10: I21, I22 |
|  |  |  |
| **Comorbidities**  **Defined from a primary or secondary in-patient or outpatient diagnosis** |  |  |
| Stroke/thromboembolism | *Defined from diagnosis of ischemic stroke, transient ischemic attack, or peripheral artery embolism* | ICD10: I63, I64, I74, G458, G459 |
| Myocardial infarction | *Defined from diagnosis* | ICD10: I21, I22 |
| Ischemic heart disease | *Defined from diagnosis* | ICD10: I20-I25 |
| Peripheral artery disease | *Defined from diagnosis* | ICD10: I70 |
| Heart failure | *Defined from diagnosis* | ICD10: I42, I50, I110, J81 |
| Diabetes mellitus | *Defined from medication for diabetes mellitus* | ATC: A10 |
| Hypertension | *Defined from treatment with at least two classes of antihypertensive drugs: adrenergic α-antagonists, non-loop diuretics, vasodilators, beta-blockers, calcium channel blockers and renin-angiotension system inhibitors* | ATC: C02A, C02B, C02C, C02L, C03A, C03B, C03D, C03E, C03X, C07B, C07C, C07D, C08G, C02DA, C09BA, C09DA, C02DB, C02DD, C02DG, C07A, C07B, C07C, C07D, C07F, C08, C09BB, C09DB, C09AA, C09BA, C09BB, C09CA, C09DA, C09DB, C09XA02, C09XA52 |
| Abnormal liver function | *Defined from diagnosis of chronic liver disease, cirrhosis and hepatitis* | ICD10: B15-B19, C22, D684C, I982, K70-K77, Q618A, Z944 |
| Previous bleeding | *Defined from diagnosis of intracranial bleeding, major gastrointestinal bleeding, respiratory or urinary tract bleeding, and bleeding due to anemia* | ICD10: D500, D62, G951A, H052A, H313, H356, H431, H450, I312, I60-I62, I850, I864A, J942, K228F, K298A, K250, K252, K254, K256, K260, K262, K264, K266, K270, K272, K274, K276, K280, K282, K284, K286, K625, K661, K638B, K638C, K838F, K868G , K920, K921, K922, N02, R04, R31, S064, S065, S066, S368D |
| Alcohol abuse | *Defined from alcohol-related diagnosis codes or at least one dispensed prescription of an alcohol antagonist drug used to treat chronic alcoholism* | ICD10: E244, E52,  F10, G312, G621, G721, I426, K292, K70, K860, L278A, O354, T51, Z714, Z721  ATC: N07BB |
| **Concomitant medication** |  |  |
| Non-steroidal anti-inflammatory drugs | *Defined from ATC-codes* | ATC: M01A without M01AX05 |
| Renin-angiotensin system inhibitors | *Defined from ATC-codes* | ATC: C09AA, C09BA, C09BB, C09CA, C09DA, C09DB, C09XA02, C09XA52 |
| Statins | *Defined from ATC-codes* | ATC: CI0AA |
| Beta-blockers | *Defined from ATC-codes* | ATC: C07A, C07B, C07C, C07D, C07F |
| Acetylsalicylic acid | *Defined from ATC-code* | ATC: B01AC06, N02BA1 |
| ADPi | *Defined from ATC-code* | ATC: B01AC04, B01AC22, B01AC24 |
