# Supplementary material for: Non-vitamin K antagonist oral anticoagulants vs. vitamin-K antagonists in patients with atrial fibrillation and chronic kidney disease: a nationwide cohort study
Source: Thromb J. 2019 Nov 12;17:21. doi: 10.1186/s12959-019-0211-y (PMC6849210; doi:10.1186/s12959-019-0211-y)
Supplement: Supplementary file 2 — Additional file 2. External analysis of kidney function in a comparable population. [file 12959_2019_211_MOESM2_ESM.docx]

**Additional file 2 – External analysis of kidney function in a comparable population**

| Estimated GFR | Number of patients |
| --- | --- |
| >90 mL/min/1.73m^2^ | 3 (0.4%) |
| 60-90 mL/min/1.73m^2^ | 31 (4.3%) |
| 30-59 mL/min/1.73m^2^ | 312 (42.9%) |
| 15-29 mL/min/1.73m^2^ | 319 (43.9%) |
| <15 mL/min/1.73m^2^ (no dialysis) | 62 (8.5%) |

*Abbreviation: GFR – Glomerular filtration rate*

*Patients had a diagnosis of atrial fibrillation and chronic kidney disease and were first-time initiators of oral anticoagulation. Plasma creatinine measurements were from general practitioner or hospital clinics. Only values within 90 days before initiation of oral anticoagulation initiation were used.*
