# Supplementary figures and images for: Non-vitamin K antagonist oral anticoagulants vs. vitamin-K antagonists in patients with atrial fibrillation and chronic kidney disease: a nationwide cohort study
Source: Thromb J. 2019 Nov 12;17:21. doi: 10.1186/s12959-019-0211-y (PMC6849210; doi:10.1186/s12959-019-0211-y)

**Additional file 3 – Cumulative incidence of events according to OAC among AF patients with CKD**

**
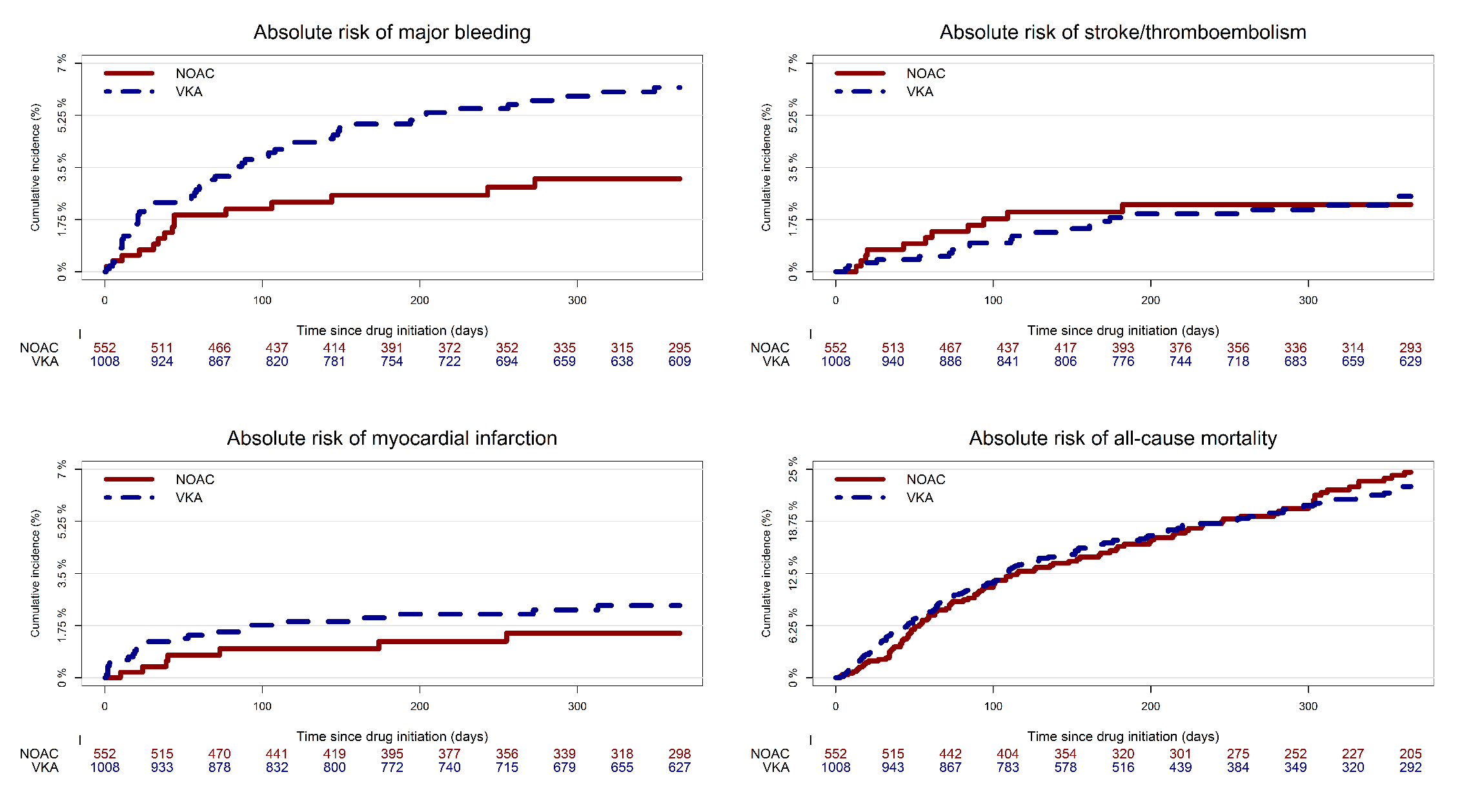
**

Supplement: Supplementary file 3 — Additional file 3. Cumulative incidence of events according to OAC among AF patients with CKD. [file 12959_2019_211_MOESM3_ESM.docx]
