# Supplementary material for: Non-vitamin K antagonist oral anticoagulants vs. vitamin-K antagonists in patients with atrial fibrillation and chronic kidney disease: a nationwide cohort study
Source: Thromb J. 2019 Nov 12;17:21. doi: 10.1186/s12959-019-0211-y (PMC6849210; doi:10.1186/s12959-019-0211-y)
Supplement: Supplementary file 4 — Additional file 4. Risk of events according to time-varying OAC. [file 12959_2019_211_MOESM4_ESM.docx]

**Additional file 4 – Risk of events according to time-varying OAC**

|  | **Number of events** | **Hazard ratio (95% CI)** |
| --- | --- | --- |
| **Stroke/thromboembolism** |  |  |
| VKA | 17 | 1.00 (reference) |
| NOAC | 4 | 0.97 (0.46-2.03) |
|  |  |  |
| **Major bleeding** |  |  |
| VKA | 47 | 1.00 (reference) |
| NOAC | 12 | 0.55 (0.31-0.95) |
|  |  |  |
| **Myocardial infarction** |  |  |
| VKA | 19 | 1.00 (reference) |
| NOAC | 3 | 0.62 (0.27-1.44) |
|  |  |  |
| **All-cause mortality** |  |  |
| VKA | 149 | 1.00 (reference) |
| NOAC | 45 | 0.91 (0.71-1.16) |

*Abbreviations: CI – confidence interval, VKA – Vitamin-K antagonist, NOAC – Nonvitamin K oral anticoagulant*
