# Supplementary material for: Non-vitamin K antagonist oral anticoagulants vs. vitamin-K antagonists in patients with atrial fibrillation and chronic kidney disease: a nationwide cohort study
Source: Thromb J. 2019 Nov 12;17:21. doi: 10.1186/s12959-019-0211-y (PMC6849210; doi:10.1186/s12959-019-0211-y)
Supplement: Supplementary file 5 — Additional file 5. Hazard ratio according to type of oral anticoagulation among atrial fibrillation patients with CKD when not censoring at shift or discontinuation of oral anticoagulation. [file 12959_2019_211_MOESM5_ESM.docx]

**Additional file 5 – Hazard ratio according to type of oral anticoagulation among atrial fibrillation patients with CKD when not censoring at shift or discontinuation of oral anticoagulation**

|  | **Number of events** | **Hazard ratio (95%CI)** |
| --- | --- | --- |
| **Stroke/thromboembolism** |  |  |
| VKA | 28 | 1.00 (reference) |
| NOAC | 15 | 0.89 (0.46-1.71) |
|  |  |  |
| **Major bleeding** |  |  |
| VKA | 68 | 1.00 (reference) |
| NOAC | 17 | 0.43 (0.25-0.73) |
|  |  |  |
| **Myocardial infarction** |  |  |
| VKA | 30 | 1.00 (reference) |
| NOAC | 7 | 0.35 (0.15-0.82) |
|  |  |  |
| **All-cause mortality** |  |  |
| VKA | 253 | 1.00 (reference) |
| NOAC | 122 | 0.89 (0.71-1.11) |

*Abbreviations: CI – confidence interval, OAC – Oral anticoagulation, VKA – Vitamin-K antagonist, NOAC – Nonvitamin K oral anticoagulant*
